# Supplementary material for: Induction of retinopathy by fibrillar oxalate assemblies
Source: Commun Chem. 2020 Jan 3;3:2. doi: 10.1038/s42004-019-0247-8 (PMC9812261; doi:10.1038/s42004-019-0247-8)
Supplement: Supplementary file 1 — Supplemental information [file 42004_2019_247_MOESM1_ESM.pdf]

Supplemental information

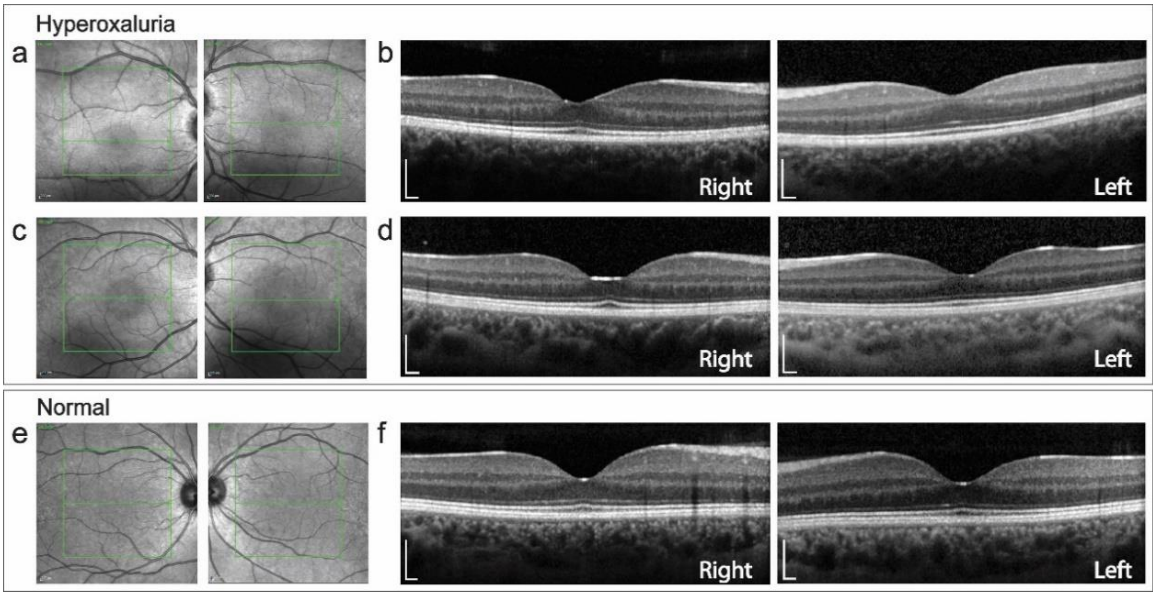

Supplementary figure 1. Absence of retinal structural abnormalities in patients with hyperoxaluria type 1. Clinical findings in (a, b) a 10-year-old patient, (c, d) a 7-year-old patient, and (e, f) a healthy subject. (a, c, e) Scanning-laser ophthalmoscopy and (b, d, f) spectral-domain optical coherence tomography (SD-OCT) images are shown, demonstrating normal macular morphology of all examined eyes. No crystal deposition or associated pathology could be detected. Similar findings were noted in all other patients.

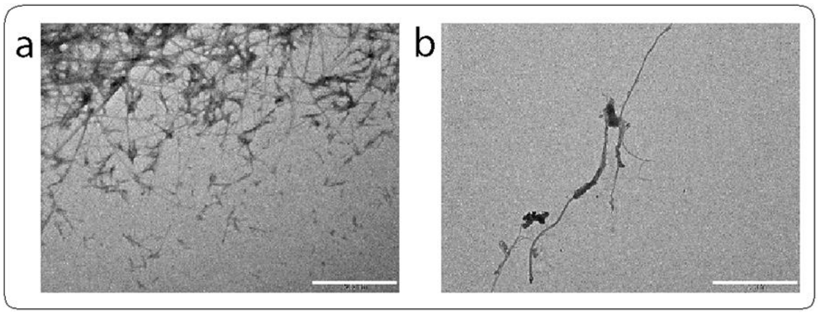

Supplementary figure 2. Sodium oxalate self-assemble into fibrils. 8 mM sodium oxalate solution in PBS was heated to 90 °C and let to cool down gradually. This resulted in the formation of fibrillar structures as visualized by TEM. (a) Sodium oxalate assemblies (scale bar: 500 nm). (b) Single sodium oxalate fibril (scale bar: 2 µm).

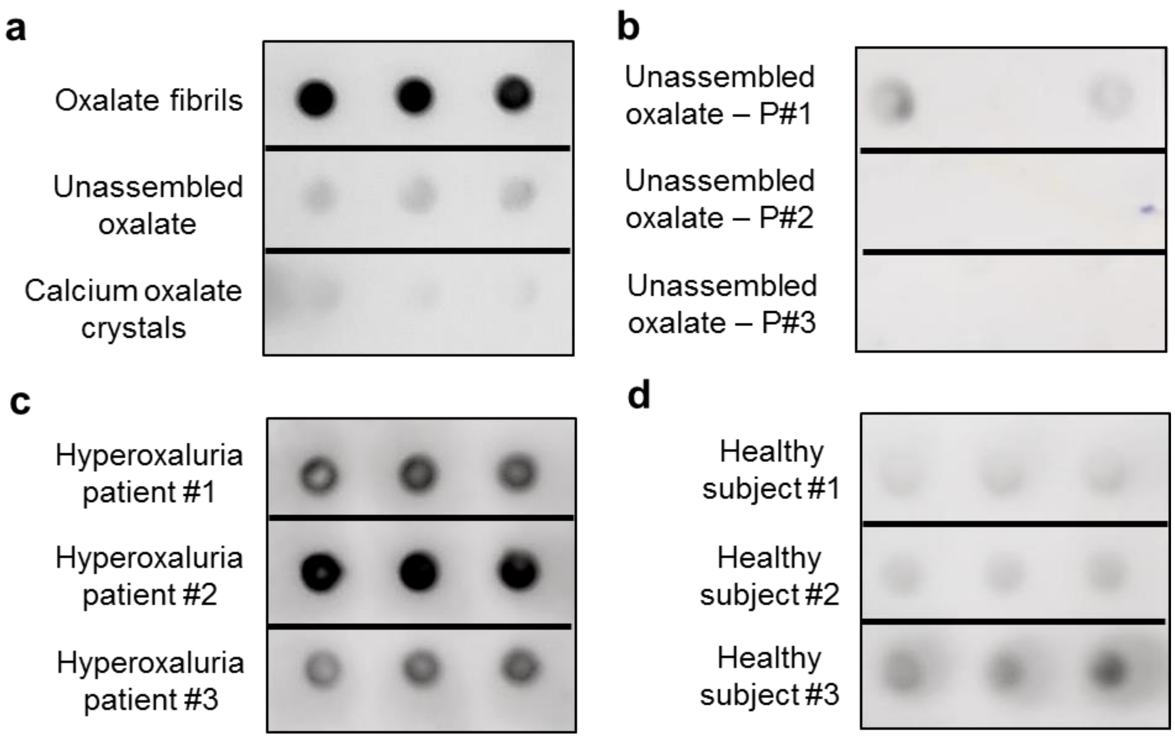

Supplementary figure 3. Anti-oxalate fibrils antibodies reactivity assay using dot blot. (a) Preformed calcium oxalate crystals, oxalate fibrils and unassembled oxalate were absorbed onto a membrane and reacted with antibodies purified from immunized rabbit sera (see methods section). Preformed oxalate fibrils presented a positive signal, while unassembled oxalate and preformed calcium oxalate crystals showed no signal. (b) Unassembled oxalate absorbed onto a membrane and reacted with the antibodies purified from the serum of different hyperoxaluria patients, resulting in a negative signal. (c) Serum of hyperoxaluria patients loaded onto a membrane and reacted with antibodies purified from immunized rabbit sera resulting in positive signal. (d) Serum of healthy subjects loaded onto a membrane and reacted with antibodies purified from immunized rabbit sera resulting in negative signal

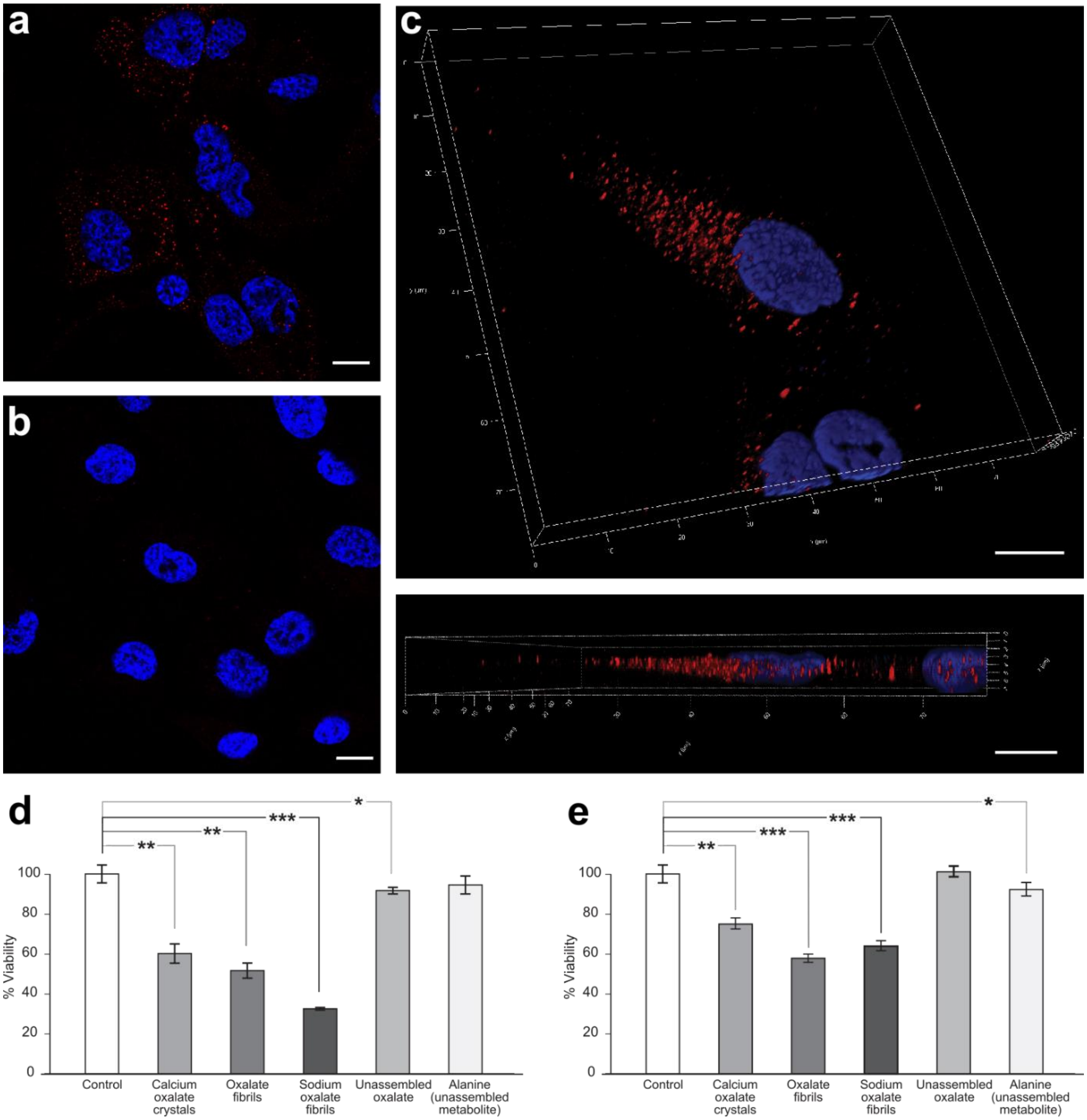

Supplementary figure 4. Immunostaining and cytotoxicity of oxalate assemblies. (a-c) ARPE-19 cells were cultured overnight with cell growth media containing oxalate fibrils, followed by immunostaining with the anti-oxalate fibrils antibodies. (a) Positive fluorescent signal was observed in the treated cells. (b) Control untreated ARPE-19 cells showed no fluorescent signal. (c) Z-stack analysis and 3D reconstruction confirmed the intracellular localization of the fluorescent signal, indicating uptake of the oxalate fibrils by the treated ARPE-19 cells. Scale bars: 100µm. (d-e) MTT cell viability assay. ARPE-19 (d) and HEK-293 (e) cells were cultured with different oxalate assemblies, unassembled oxalate and with the un-aggregative amino acid alanine, all in cell growth media, as indicated (see Methods section). The control reflects cell growth media with no oxalate, which was cultured in the same manner. Following an overnight incubation, the MTT reagent was added for 4 hours at 37 °C. Then, a detergent solution was added for another 30 min incubation at 37 °C. Absorbance was determined at 570 nm and 680nm. The results represent three biological repeats; the data are presented as mean ± SD. All statistics are in relation to the control, student's t-test * $p<0.05$, ** $p<0.01$, *** $p<0.001$.

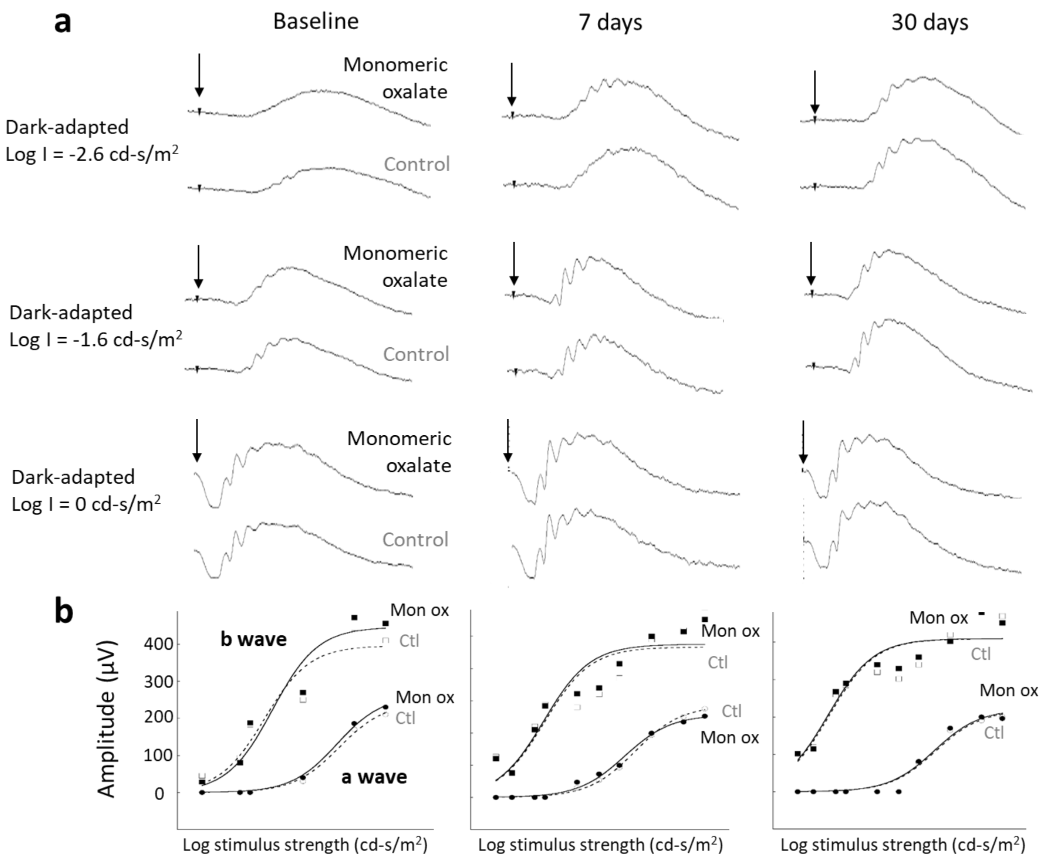

Supplementary figure 5. Unassembled oxalate has no toxic impact on retinal function. (a) Dark-adapted ERG responses elicited by flashes of different strengths, as denoted in log units to the left, are shown for each recording session. Arrow: time of light stimulus. (b) Response-stimulus strength relationships for the dark-adapted ERG responses of the same rat. The curves were fitted to a hyperbolic type function. Overall, no deleterious effect on retinal function was noted in rat eyes treated with intravitreal administration of monomeric oxalate. Similar findings were noted in all other study rats (n=3).
